# Analysis of Occupational Accidents in the Spanish Mining Sector in the Period 2009–2018

**DOI:** 10.3390/ijerph182413122

**Published:** 2021-12-12

**Authors:** Lluís Sanmiquel, Marc Bascompta, Josep M. Rossell, Hernan Anticoi

**Affiliations:** 1 Department of Mining Engineering-Industrial and ICT, Polytechnic University of Catalonia, 08242 Manresa, Spain; marc.bascompta@upc.edu; 2 Department of Mathematics, Polytechnic University of Catalonia, 08242 Manresa, Spain; josep.maria.rossell@upc.edu; 3 Department of Transportation and Process Project Technology, Universidad de Cantabria (UNICAN), Boulevard Ronda Rufino Peón, 254, 39316 Torrelavega, Spain; anticoihf@unican.es

**Keywords:** accident types, deviation, physical activity, incidence risk index, severity risk index

## Abstract

Occupational accidents in the Spanish mining industry have been substantially reduced over the last decades. However, the incidence rate shows higher values than other leading mining countries. In this regard, the research carried out reveals the factors influencing the high incidence rates of the Spanish mining sector, based on three scenarios: underground mining (UG), quarries and open pit mining (OP) and mineral processing plants (PP). The three most common types of accident for each scenario have been determined, considering the accidents in Spain between 2009 and 2018. The analysis also includes the main deviations, and physical activities that the injured worker was carrying out at the time of the accident. Besides, a model to predict the number of accidents based on the lost working days is also presented together with the incidence and severity risk index adjusted by the number of employees and their worked hours, respectively, in each scenario. These finding can be relevant to define the most effective measures and policies to reduce the number of accidents in the mining sector.

## 1. Introduction

Accidents and occupational diseases can produce important direct and indirect costs to the society as a whole. While direct costs are easier to quantify, they represent a lower proportion than indirect costs [1], which can remain hidden within each organization. These costs are formed by injuries of different degrees and fatalities, as well as economic losses. In this regard, there is a direct relationship between countries with a low ratio of occupational accidents and higher levels of competitiveness [2].

The evolution of health and safety (H&S) conditions in Spain has been quite relevant in the last number of decades, especially after the framework established by the Labor Risk Prevention Act (Law 31/1995), which is the transposition of European Directive 89/391/CEE. Its application constituted the principles to improve the H&S conditions in any company and the rights and responsibilities of each stakeholder. In this sense, the obligation, and standardization, to analyze the incidents and accidents boosted the continuous improvement of all the organizations.

Several studies point out the mining sector as a dangerous economic activity [3,4,5,6,7], revealing the conditions that have an influence on the severity and number of accidents. Thus, the main ones are the environmental conditions, with a significant presence of dust, falling rocks, high degree of humidity and fixed and mobile machinery. Besides, an inadequate H&S management can involve a higher negative effect to workplace accidents than in other economic sectors with better environmental conditions. Likewise, as other industries, such as construction, the workers play a key role in many mining workplaces. Thus, some human-related factors, such as the physical and mental health of the workers, can play a pivotal role in occupational safety too [8].

The correct management involve any possible characteristic related to the workforce available and the workplace conditions. In this way, research conducted in an open pit mine from Turkey concluded that young workers must be trained and workers with more experience should perform the most critical jobs, finding the higher frequency of accidents in the range of 18 to 30 years [9]. A study performed on truck-related fatal accidents in surface mining showed that the two most common causes of these accidents are pre-operational improper check and poor maintenance. Furthermore, the non-use of seat belts and inadequate training were also two important factors [10]. An analysis of the high frequency of accidents associated with the non-powered hand-tool use has been observed in the mining sector. It concluded that mining companies should develop new and creative approaches to train employees and control these risks in order to keep reducing the incidence rate [11]. Problems related to inadequate risk prevention management systems have also been found in previous research [12,13,14,15], while changes in H&S regulations, based in the attitude towards the safety, led to a significant reduction in fatal injuries in Serbian underground coal mines [16]. The lack of a proper legislative framework and procedures have also been found as important sources of accidents in several countries [17,18]. Hence, correct H&S management requires recorded information, either internally by a company or by a government entity. The data collected can be used to define patterns and weaknesses and, subsequently, apply improvements or specific actions to tackle the issue. However, there is still a long way to go to improve the H&S conditions and the related data collected.

The overall Spanish economic sectors recorded a lost time injury frequency rate (LTIFR) of 22 workdays lost per one million of worked hours in 2018. While, the LTIFR in the Spanish mining sector was 62.8, which is 2.9 times higher than the Spanish global value. Besides, rates from the Spanish mining sector are also considerably higher than some other countries such as USA and the State of Queensland (Australia), having a rate of 8.3 to 19 respectively.

The aim of this research is to investigate several factors and characteristics of the occupational accidents and diseases in the Spanish mining sector using several approaches and find some patters that could help to reduce the occupational accident rate. The study was carried out by means of a classification of the Spanish mining industry into 3 types of mining: underground mining (UG), quarries and open pit mining (OP) and mineral processing plants (PP).

## 2. Materials and Methods

### 2.1. Study Population

2731 mining activities were active in Spain in 2018, with the following direct employment: 3905 (16.3%) in UG, 13,122 (54.8%) in OP and 6906 (28.9%) in PP. The population-based study is constituted by the occupational accidents recorded in the Spanish mining sector during the period 2009–2018. The accidents are classified in three different places: UG, OP and PP. Data was obtained from the annual digital database on accidents of the Spanish Ministry of Labor and Social Economy, using the software ArcGis v10.3 (Esri Geospatial Solutions Spain S.L, Madrid, Spain). The accidents considered in the study are those that took place in mining work center, within regular work hours (accidents on the way to/from work were not considered), and which caused the injured worker to miss at least one workday. Thus, the injuries were 16,985 in UG; 5659 in OP and 7784 in PP during the period 2009–2018.

### 2.2. Methods

The methodology used by the Spanish Ministry of Labor and Social Economy to feed the accidents digital database has been adopted to classify and codify the type of accident and the deviations. According to the Ministry, a deviation is an abnormal occurrence that has adversely interfered with the normal process of performing the work and it has directly led to the occurrence or origin of the accident.

Different studies already published focused on occupational accidents in the mining industry have employed the average lost workdays as a measure to determine risk indexes of work posts and tasks, among groups of workers, and types of mines [19,20,21,22]. This measure enables one to evaluate the overall safety performance within a specific mine, since it can reflect factors, such as the effective use of first aid and rapid access to medical care, the use of personal protective equipment, and company’s policies concerning return-to-work practices [23]. In order to take account of the fatal accidents with a standard value of 6000 lost workdays, we modelled the distribution that was more closely in accordance with the number of accidents, considering the number of lost workdays both in underground coal, metal and non-metal mines, using the following density function detailed in Equation (1).

(1) $$f\left(x\right)=\frac{1}{b}e^{\frac{-\left(x-\theta \right)}{b}}\;,\;x>\theta \;,\;b>0$$

The fatal accidents are not considered in this study due to their low frequency. In this way, the relative percentage of the fatal accidents was 0.15% in UG, 0.58% in OP and 0.30% in PP. The best function that fits a set of points (*x*, *y*) has been found, where *x* denotes the lost workdays and *y* the number of injuries for each scenario (UG, OP and PP). The scatter diagram of the points (*x*, *y*) shows a potential good fit with an exponential function, $y=ae^{bx}$, where a, b are the parameters to be calculated. The model can be linearized in the form  lny=lna+bx and, using the method of least squares, obtain the best fit for the parameters a and b together with the coefficient of determination, $R^{2}$, which provides the goodness of the fitted model [24]. Minitab v19 was used to obtain the best linear function that fits the set of points.

The risk index was also calculated, adjusted by the workplace size, center size, of the owner company of the mining activity, in number of workers per each place (UG, OP and PP). Six groups were established for the variable size: 1–9, 10–19, 20–49, 50–99, 100–499, ≥500. The risk index is indicative of the incidence of accidents among different groups or subpopulations [25], and it is defined as the ratio of percentage of injured workers, of a given subpopulation, to the percentage of the total workforce represented by this subpopulation (Equation (2)). The same concept can also be used to find a risk index which is an indicator of the severity of accidents (Equation (3)).

(2) $$Incidence\;Risk\;Index=\frac{Accidents\;\left(\%\right)}{Workers\;\left(\%\right)}$$

(3) $$Severity\;Risk\;Index=\frac{\%Lost\;Workdays}{\%\;Worked\;Hours}$$

A risk index = 1 corresponds to an average incidence or severity rate of work-related accidents, whereas a value greater than 1 indicates a higher risk (incidence or severity) for the group and a value smaller than 1 means a lower incidence or severity.

The percentage of occupational accidents and the lost workdays, for the six sizes of company groups defined in the previous paragraph, was obtained from the annual digital database of accidents from the Spanish Ministry of Labor and Social Economy, using the software ArcGis v10.3. The percentage of workers and worked hours by size of company group was obtained from the annual mining statistics of the Spanish Ministry for Ecological Transition and Demographic Challenge.

## 3. Results and Discussion

### 3.1. Accident Analysis

The three most common types of occupational accident in UG, OP and PP were determined from the analysis of data sources for the period 2009–2018, Table 1. While the main characteristics of these accidents, regarding severity, are detailed in Table 2.

The results obtained regarding the most common type of accidents in UG, OP and PP are similar, especially in OP and PP, where they have the same three types of most frequent accident. Thus, the most common type in all three was #71 (physical over-exertion on the muscular-skeletal system). In UG it is followed in second place by #42 (to be hit by a falling object or one that is detached) and in third place by #31 (blows or hitting something as a result of a fall). This last one is also the second most common in OP and PP. Whereas the third place is also shared by OP and PP, #32 (Blows as the result of a fall, or crashing into an immovable object). An important fact is that the second and third most frequent types of accidents in OP and PP are related to moving objects, the injured person collides or hits against it.

**Figure 1:**
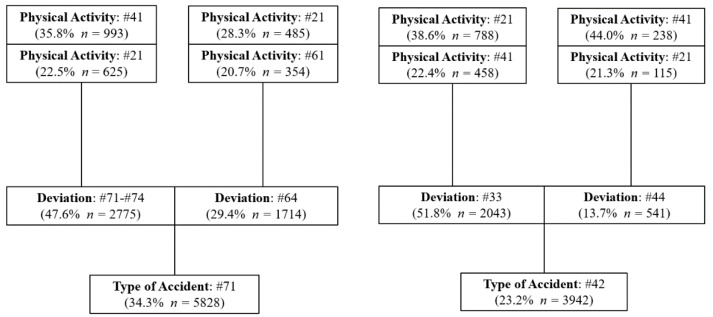
Characteristics of the Type of Accident in UG.

**Figure 2:**
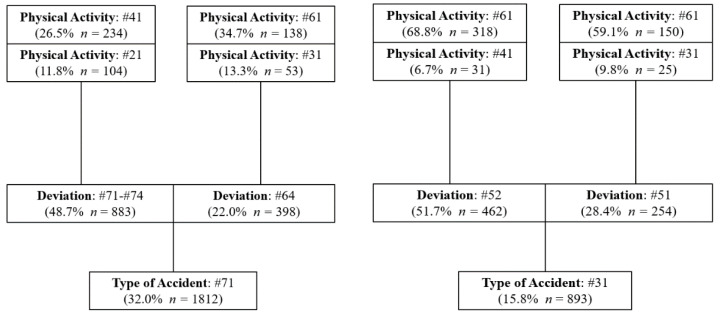
Characteristics of the Type of Accident in OP.

**Figure 3:**
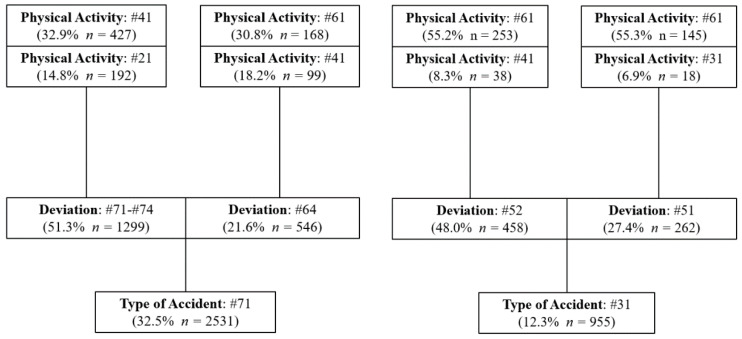
Characteristics of the Type of Accident in PP.

Figure 1, Figure 2 and Figure 3 show for each of the two most frequent types of accidents in the three types of mining activities analyzed (UG, OP and PP), the two main deviations that influenced the genesis of the accident and the two most relevant physical activities that the injured workers were carrying out at the time of the accident.

Regarding the most important characteristics of the accidents that have occurred in the three scenarios, the most common accident is the code #71, preceded in all cases by a deviation, predominantly of code #71-#74, which refers to the movement of the worker’s body as a result of physical exertion due to the handling of a load or object. This type of deviation has accounted for 47.6% in UG, 48.7% in OP and 51.3% in PP. Therefore, a preventative policy focused on reducing this type of deviation could lead to a significant reduction in accidents.

The most frequent physical activity performed by the worker at the time of these accidents was code #41 (manipulation of objects: to take by hand, grasp, hold, put-in a horizontal plane) which occurred 35.8% in UG, 26.5% in OP and 32.9% in PP. While the second most frequent physical activity was code #21 (working with non-powered hand tools). Therefore, a greater mechanization of tasks in these workplaces could imply a reduction of the type of accident #71 with a deviation #71–#74. A more intense training policy concerning the proper handling of loads and objects could also contribute to a reduction of these accidents.

The second most important deviation for accidents with code #71 in the three scenarios was code #64 (movement of the worker’s body without added physical effort. uncoordinated movements, untimely or inopportune gestures). The most frequent physical activity performed by the worker at the time of the accident was code #21 (working with non-powered hand tools) and #61 (worker movements: walk, run, jump, stand up, sit down...) in UG. On the other hand, the predominant physical activity was code #61 in OP and PP. The second most frequent physical activity was code #31 (driving a transport vehicle or loading equipment-mobile and motorized) in OP and code #41 (manipulation of objects: to take by hand, grasp, hold, put-in a horizontal plane) in PP.

Regarding other types of accident, code #42 is found in UG, where more than half of them (51.8% *n* = 2043) were preceded by a deviation code #33 (slip, fall, collapse of an upper material agent falling on the injured worker). It is particularly important to consider preventive measures to minimize this type of accident, such as training workers on inspections and workplace control procedures [26] since it can lead to severe accidents and even fatalities. Besides, an increase in the analysis of incidents and accidents incidents related to falling roofs and sidewalls could provide more information on these types of accidents and, consequently, be able to adopt more effective preventive measures, such as: drift supervision, support design, convergence control, etc.

In OP and PP, the second most important accident is code #31 (Blows or collisions against something as a consequence of a fall of the worker), with the two most frequent deviations related to the fall of the worker, at the same level (#52), and from a height of more than 2 meters (#51). The deviation due to falls at the same level was much more frequent than the fall from a height in OP and PP, 51.7% and 48.0% compared to 28.4% and 27.4%, respectively. These data suggest that a significant reduction in these accidents could be achieved by applying preventive measures aimed at: improving the order and cleanliness of workplaces in OP and PP, as well as keeping the widths of walkways and passageways in good conditions in PP. The physical activity performed by the worker injured in the accident form #31 is the same for OP and PP, highlighting the physical activity #61 (worker movements: walk, run, jump, stand up, sit down, etc.).

The results obtained also show some interesting differences respect to previous research. A study of fatal electrical accidents in Australia [27] found that 70% of these accidents had immediate causes attributable to workplace environmental conditions. Besides, additional studies on occupational accidents recorded in the Spanish mining industry reveal that approximately 67% of serious and fatal accidents occurring in UG and 51% of accidents recorded in OP and PP had an immediate cause directly related to environmental conditions [7]. However, outcomes from the present study only indicate that 37.3% (*n* = 6343) of the accidents occurring in UG, 36.8% (*n* = 2082) in OP and 32.1% (*n* = 2496) in PP had a deviation related to environmental workplace conditions. It is believed that these differences are attributed to the fact that the analysis in this study consider minor and serious accidents, whereas the two previous studies only considered fatal accidents [27] or serious and fatal accidents [7]. If the analyses were done with only serious and fatal accidents, the percentage would be much closer in UG, 65.8% (73 out of 111), but not in OP with 62.6% (107 out of 171) and PP with 41.2% (54 out of 131). These differences are caused by the fact OP and PP are analyzed together in [7], reaching a 53.3% (161 out 302) if the same approach is applied, which gives a similar result.

### 3.2. Analysis of the Distribution of the Number of Accidents Based on the Lost Workdays

**Figure 4:**
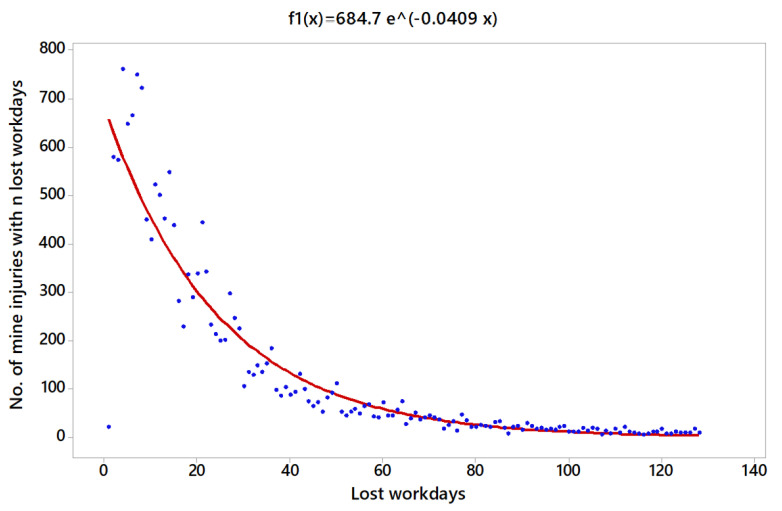
Regression model for the number of UG injuries.

The injuries are obtained by the following linear model: lny=6.529−0.0409 x, where a=684.7 and b=−0.0409. Figure 4 shows the fitted exponential function denoted by $f_{1}\left(x\right)=684.7e^{-0.0409\;x}$. Where 1 is the minimum and 128 is the maximum number of lost workdays registered in the data base, $\int_{1}^{128}f_{1}\left(x\right)dx\;$= 15981.0. Dividing $f_{1}\left(x\right)$ by this value, it is possible to obtain a new function $p_{1}\left(x\right)=0.00428\;e^{-0.0409\;x}$, which can be used as a probability density function. A $R^{2}=88.1\%$ was obtained, which can be considered as a good coefficient of determination for predictions.

**Figure 5:**
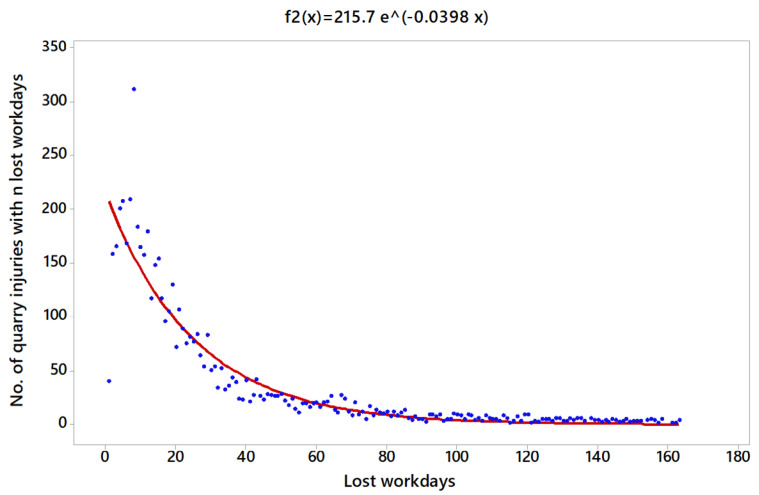
Regression model for the number of OP injuries.

The obtained linear model for OP is given by lny=5.374−0.0398 x, with a high coefficient of determination, $R^{2}=84.8\%$. The fitted regression function is $f_{2}\left(x\right)=215.7e^{-0.0398\;x}$ (Figure 5), with $\int_{1}^{163}f_{2}\left(x\right)dx=5199.88$. Thus, $p_{2\;}\left(x\right)=0.041e^{-0.0398\;x}$ can be used as a probability density function.

**Figure 6:**
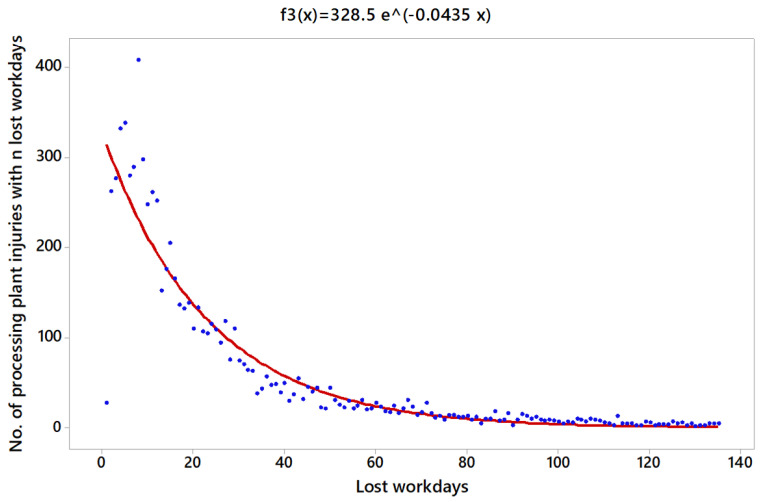
Regression model for the number of PP injuries.

On the other hand, the function for PP injuries is lny=5.795−0.0435x, with $R^{2}=89.4\%$, which is the best fit of the three scenarios. Then, $f_{3}\left(x\right)=328.5\;e^{-0.0435\;x}$ (Figure 6). Furthermore, $\int_{1}^{135}f_{3}\left(x\right)\;dx=7209.0$ and the corresponding probability density function was $p_{3\;}\left(x\right)=0.045\;e^{-0.0435\;x}$.

In addition, Table 3 shows the probability of an accident involving more than 10, 20, 30 and 60 days off work in each scenario for the whole period analysed, 2009–2018. The accidents located in OP registered the most severe consequences, whereas PP accounted for the least severe consequences.

Despite the values obtained are quite similar in the three scenarios. The worst case regarding the severity based on workdays lost is for OP, followed by UG and PP, respectively.

The models proposed allow for a very direct comparison of the accidents severity for different groups, companies, economic sectors, types of accidents, type of mining site (UG, OP and PP in this case), etc. This information allows one to carry out the necessary analyses and investigations in order to discover the most relevant features in each scenario, facilitating the adoption of more effective corrective measures. In this regard, Table 3 shows that accidents in PP had a lower average duration of sick leave than in UG and OP.

A deeper and more detailed analysis focused on the differences between PP and OP should be performed, perhaps by modelling each of the most frequent types of accidents and deviations in OP and PP. This could allow one to identify the main differences in each type of accident.

### 3.3. Incidence and Severity Risk Index Based on the Center Size

The incidence risk index is analyzed considering the percentage of accidents and the workers in the pre-established centers size by number of employees, considering the three scenarios: UG, OP and PP (Table 4, Table 5, Table 6, Table 7, Table 8 and Table 9).

While UG have a center size and gather the majority of the accident at the top of the range, OP and PP are the opposite. This fact is due to OP and PP include all the quarries and open pit mines, having a large group of small activities, which in many cases have a processing plant as well. On the other hand, UG require large infrastructure and investment, being most common to have a large activity.

The incidence risk index, percentage of the number of accidents as a function of the percentage of the number of workers, is considerably higher in UG than in OP and PP, having the last two very similar values. In UG and OP, the highest incidence of accidents occurs in the center size of 20–49 workers, while in PP it occurs in those plants with 50–99 workers. Likewise, the lowest incidence of accidents occurs in centers with more than 500 workers.

The severity risk index is analysed by considering the percentage of lost workdays and the worked hours in the pre-established centres size by number of employees, and by considering the three scenarios: UG, OP and PP (Table 8, Table 9 and Table 10).

As for the severity risk index, percentage of the number of days lost as a function of the percentage of hours worked, is much higher in UG than in OP and PP, which are relatively equal. This coincides with the incidence risk index, as well as the maximum and minimum values for the three scenarios analyzed. All these accident incidence and severity data expressed in this study as risk indexes confirm previous studies [3,4,5,6,7], in which the more unfavorable and dangerous environmental conditions are found in the UG scenario.

Another aspect to be considered is that the three regression functions obtained, Figure 4, Figure 5 and Figure 6, are indicative of the average duration of sick leave in accidents. On the other hand, the severity risk index obtained in Table 11 is directly related to the severity of accidents as a function of the number of absences in relation to the number of hours worked. This leads to some, apparently, contradictory aspects, such as that accidents in OP are more serious if the average duration of the sick leave is analyzed, followed by PP and UG. However, if the days lost are studies in relation to the number of hours worked, the results indicate that the accidents with the most serious consequences were those in UG, followed by PP and finally OP.

In other words, the results differ depending on whether one takes into account the ratio of days lost per number of hours worked, or the ratio of days lost per number of accidents. A plausible explanation for this is that the severity index and the average duration index express the severity of accidents at work differently, which may mean that their trends (downward or upward) may or may not coincide in the comparison between different sectors. Being necessary to analyze separately the average duration of sick leave and the ratio of the number of sick leaves to the hours worked.

The limitations of the study derive fundamentally from the accident database itself. Thus, according to the accidents, data is not possible to determine whether an accident is attributable to human error or to some deficiencies in the preventive organization. This fact is caused by a lack of enough information to know the exact circumstances immediately prior to the accident. Moreover, it is impossible to know all the details of the basic causes that have led to the presence of immediate causes and, subsequently, the occurrence of a deviation. Further research is necessary, with new data, in order to know the evolution of the accident’s behavior over time as a sector and, depending on the type of mining activity (OP, UG and PP), policies implemented and inclusion of new features in the accident’s database.

## 4. Conclusions

The most common types of accidents have been determined in UG, OP and PP, sharing two of the three most common types in the three scenarios, while OP and PP have the same type and order, slightly differing only in its relevance. Besides, the last two most frequent types of accidents in OP and PP are related to the fact that the injured person collides or hits against a moving object. On the other hand, the knowledge of the most common type of accident and deviation could allow one to define the most effective policy to reduce the number of accidents. Thus, preventive measures focused on the reduction of accidents of type #71, deviation #71–#74, are the ones that can lead to a greater reduction of the occupational accident rate. Special attention should also be given to UG accidents with type #42, deviation #33, due to its high incidence.

The distribution function in the three scenarios (UG, OP and PP), without including the fatalities, gives a coefficient of determination of at least almost 85%, considering the models adequate to predict the number of accidents based on the lost working days in all the scenarios proposed. In this regard, the severity based on the workdays lost is higher in OP, followed by UG and PP, respectively.

The incidence risk index shows that the mining activities usually have a better H&S system when the organization is bigger, than in smaller activities. The highest index is found in middle size activities in the three scenarios. In addition, the incidence risk index and the severity risk index show much higher values in UG than in OP or PP, increasing substantially the incidence and severity index of the mining sector. Further research is required to determine the specific reason of this behavioral pattern.

## Figures and Tables

**Table 1:** Distribution per type of accident recorded in the UG, OP and PP.

Type of Accident Code
UG (16,985 accidents)
#71: Physical over-exertion on the muscular-skeletal system (34.3%) #42: To be hit by a falling object or one that is detached (23.2%) #31: Blows or hitting something as a result of a fall (8.1%)
OP (5659 accidents)
#71: Physical over-exertion on the muscular-skeletal system (32.0%) #31: Blows or hitting something as a result of a fall (15.8%) #32: Blows as the result of a fall, or crashing into an immovable object (9.6%)
PP (7784 accidents)
#71: Physical over-exertion on the muscular-skeletal system (32.5%) #31: Blows or hitting something as a result of a fall (12.3%) #32: Blows as the result of a fall, or crashing into an immovable object (9.2%)

**Table 2:** Description of the Deviation Codes and Physical Activity Codes.

**Deviation Code**
#71–74: Worker’s body movement as a result of physical exertion due to handling a load or object
#64: Worker’s body movement without added physical effort. Uncoordinated movements, untimely or inopportune gestures #33: Slip, fall or collapse of an upper material agent (that falls on the injured worker) #44: Loss (total or partial) of control of the object or material (transported, moved, handled, etc.) #52: Fall of a person-to the same level #51: Fall of a person-from a height
**Physical Activity Code**
#61: Worker movements: walk, run, jump, stand up, sit down, etc. #41: Manipulation of objects: to take by hand, grasp, hold, etc. -in a horizontal plane #31: Driving a vehicle or loading equipment -mobile and motorized #21: Working with non-powered hand tools

**Table 3:** Probability of an accident involving different lost workdays.

	UG	OP	PP
≥10 lost workdays	69.0%	69.8%	67.5%
≥20 lost workdays	45.7%	46.9%	43.6%
≥30 lost workdays	30.1%	31.4%	28.1%
≥60 lost workdays	8.4%	9.4%	7.4%

**Table 4:** Workers and accidents per size in UG (%).

	**Center Size**					
**Year**	**1–9**	**10–19**	**20–49**	**50–99**	**100–499**	**≥500**
**2009**	0.23	0.24	2.58	4.18	41.12	51.65
**2010**	0.74	0.88	2.71	5.98	36.55	53.13
**2011**	0.34	1.00	3.46	8.30	42.14	44.75
**2012**	0.41	1.44	4.90	4.19	38.93	50.14
**2013**	0.55	1.13	1.37	6.28	39.63	51.04
**2014**	0.65	1.04	4.98	2.91	52.21	38.22
**2015**	0.36	1.20	3.06	3.44	41.83	50.10
**2016**	0.11	1.60	2.57	3.06	26.83	65.83
**2017**	0.08	1.92	3.30	3.22	28.72	62.76
**2018**	0.36	1.58	2.54	1.45	24.85	69.23
**Average**	0.39	1.14	3.12	4.47	37.75	53.13
**Accidents**						
**Year**	**1–9**	**10–19**	**20–49**	**50–99**	**100–499**	**≥500**
**2009**	0.82	1.40	11.68	12.37	59.60	14.13
**2010**	1.06	2.40	10.72	13.24	59.31	13.28
**2011**	0.59	0.98	11.81	6.10	67.35	13.18
**2012**	0.67	1.89	15.21	10.27	67.15	4.83
**2013**	1.00	2.46	10.15	10.22	71.07	5.11
**2014**	0.73	2.31	9.83	13.35	68.99	4.79
**2015**	1.59	1.53	13.26	13.79	65.12	4.71
**2016**	1.28	2.30	12.94	8.09	69.87	5.53
**2017**	0.87	1.25	12.72	7.23	70.57	7.36
**2018**	0.90	1.47	9.84	4.98	73.76	9.05
**Average**	0.92	1.80	11.79	10.63	65.68	9.18

**Table 5:** Workers and accidents per size in OP (%).

	**Center Size**					
**Year**	**1–9**	**10–19**	**20–49**	**50–99**	**100–499**	**≥500**
**2009**	41.96	25.30	18.91	6.86	6.96	
**2010**	45.13	25.74	17.83	6.69	4.61	
**2011**	45.69	25.62	17.34	7.31	4.05	
**2012**	49.07	24.62	15.77	6.58	3.97	
**2013**	48.04	23.51	17.57	6.15	4.74	
**2014**	49.82	23.31	17.62	5.66	3.59	
**2015**	48.14	22.79	18.38	7.49	3.20	
**2016**	43.60	23.19	17.63	6.70	8.89	
**2017**	42.48	24.03	17.51	6.81	9.17	
**2018**	40.78	25.64	18.57	6.86	8.15	
**Average**	45.22	24.50	17.76	6.73	5.78	
**Accidents**						
**Year**	**1–9**	**10–19**	**20–49**	**50–99**	**100–499**	**≥500**
**2009**	26.03	28.64	27.84	10.05	7.14	
**2010**	27.41	28.56	27.98	9.06	6.77	
**2011**	29.23	26.93	26.22	7.45	10.03	
**2012**	36.06	26.69	22.31	6.37	8.57	
**2013**	32.80	31.18	22.31	6.45	6.99	
**2014**	42.30	24.83	19.31	4.83	8.51	
**2015**	32.23	25.39	26.49	7.73	8.17	
**2016**	35.75	25.85	30.19	3.38	4.59	
**2017**	38.20	24.33	24.82	5.84	6.33	
**2018**	32.54	24.65	30.18	5.92	6.11	
**Average**	31.90	26.98	26.21	7.26	7.40	

**Table 6:** Workers and accidents per size in PP (%).

	**Center Size**					
**Year**	**1–9**	**10–19**	**20–49**	**50–99**	**100–499**	**≥500**
**2009**	29.18	17.62	13.91	6.04	17.43	15.83
**2010**	31.94	18.36	13.34	6.48	12.78	17.11
**2011**	31.19	17.75	12.90	7.62	14.53	16.01
**2012**	33.06	16.99	12.19	5.79	13.50	18.46
**2013**	32.15	16.02	12.15	6.19	14.24	19.24
**2014**	33.83	15.99	13.23	4.48	14.33	18.14
**2015**	33.57	16.20	13.71	6.26	12.63	17.63
**2016**	32.16	17.57	13.77	5.86	8.56	22.08
**2017**	31.68	18.47	14.01	6.01	7.30	22.54
**2018**	31.57	20.20	15.00	5.67	7.10	20.46
**Average**	31.98	17.54	13.41	6.07	12.37	18.64
**Accidents**						
**Year**	**1–9**	**10–19**	**20–49**	**50–99**	**100–499**	**≥500**
**2009**	15.36	17.85	22.56	17.93	22.85	3.45
**2010**	17.95	16.79	22.36	18.71	21.69	2.50
**2011**	18.37	17.50	20.43	20.54	19.46	3.70
**2012**	19.94	18.35	16.18	20.23	19.80	5.49
**2013**	20.70	18.36	21.37	20.20	14.69	4.67
**2014**	21.34	18.49	18.32	19.33	17.98	4.54
**2015**	22.79	16.79	15.59	15.59	22.94	6.30
**2016**	21.07	16.35	23.43	16.04	16.67	6.45
**2017**	21.81	16.70	25.38	13.97	13.97	8.18
**2018**	18.10	16.79	25.84	14.89	15.62	8.76
**Average**	21.07	16.35	23.43	16.04	16.67	6.45

**Table 7:** Incidence risk index per size and each scenario.

**UG**						
**Year**	**1–9**	**10–19**	**20–49**	**50–99**	**100–499**	**≥500**
**2009**	3.56	5.82	4.53	2.96	1.45	0.27
**2010**	1.43	2.71	3.95	2.21	1.62	0.25
**2011**	1.70	0.97	3.41	0.74	1.60	0.29
**2012**	1.62	1.31	3.10	2.45	1.73	0.10
**2013**	1.82	2.17	7.42	1.63	1.79	0.10
**2014**	1.12	2.22	1.97	4.59	1.32	0.13
**2015**	4.40	1.27	4.33	4.01	1.56	0.09
**2016**	11.20	1.44	5.04	2.64	2.60	0.08
**2017**	11.04	0.65	3.85	2.24	2.46	0.12
**2018**	2.54	0.93	3.88	3.44	2.97	0.13
**Average**	2.37	1.57	3.78	2.38	1.74	0.17
**OP**						
**Year**	**1–9**	**10–19**	**20–49**	**50–99**	**100–499**	**≥500**
**2009**	0.62	1.13	1.47	1.47	1.02	
**2010**	0.61	1.11	1.57	1.35	1.47	
**2011**	0.64	1.05	1.51	1.02	2.47	
**2012**	0.73	1.08	1.41	0.97	2.16	
**2013**	0.68	1.33	1.27	1.05	1.48	
**2014**	0.85	1.07	1.10	0.85	2.37	
**2015**	0.67	1.11	1.44	1.03	2.55	
**2016**	0.82	1.11	1.71	0.51	0.52	
**2017**	0.90	1.01	1.42	0.86	0.69	
**2018**	0.80	0.96	1.63	0.86	0.75	
**Average**	0.71	1.10	1.48	1.08	1.28	
**PP**						
**Year**	**1–9**	**10–19**	**20–49**	**50–99**	**100–499**	**≥500**
**2009**	0.53	1.01	1.62	2.97	1.31	0.22
**2010**	0.56	0.91	1.68	2.89	1.70	0.15
**2011**	0.59	0.99	1.58	2.70	1.34	0.23
**2012**	0.60	1.08	1.33	3.49	1.47	0.30
**2013**	0.64	1.15	1.76	3.26	1.03	0.24
**2014**	0.63	1.16	1.38	4.31	1.26	0.25
**2015**	0.68	1.04	1.14	2.49	1.82	0.36
**2016**	0.66	0.93	1.70	2.74	1.95	0.29
**2017**	0.69	0.90	1.81	2.32	1.91	0.36
**2018**	0.57	0.83	1.72	2.62	2.20	0.43
**Average**	0.66	0.93	1.75	2.64	1.35	0.35

**Table 8:** Worked hours and lost workdays per size in UG (%).

	**Worked Hours**					
**Year**	**1–9**	**10–19**	**20–49**	**50–99**	**100–499**	**≥500**
**2009**	0.25	0.36	3.16	5.12	46.17	44.94
**2010**	0.61	0.96	2.76	5.32	32.34	58.01
**2011**	0.29	1.04	3.02	5.91	35.19	54.55
**2012**	0.27	1.30	4.47	3.73	33.99	56.23
**2013**	0.43	0.87	1.32	3.48	32.09	61.81
**2014**	0.48	1.14	6.63	2.63	58.84	30.28
**2015**	0.32	0.96	3.04	2.96	33.29	59.43
**2016**	0.15	1.51	2.61	2.42	19.24	74.06
**2017**	0.07	1.38	2.65	1.22	18.43	76.25
**2018**	0.29	1.42	2.94	1.42	17.30	76.62
**Average**	0.32	1.07	3.24	3.53	33.15	58.69
**Workdays Lost**						
**Year**	**1–9**	**10–19**	**20–49**	**50–99**	**100–499**	**≥500**
**2009**	2.63	1.58	13.50	12.51	54.13	15.65
**2010**	1.58	3.35	14.95	15.65	51.34	13.13
**2011**	0.19	1.32	10.42	5.74	71.45	10.89
**2012**	0.58	2.37	14.32	9.02	69.81	3.89
**2013**	0.61	2.23	7.70	9.39	76.68	3.40
**2014**	0.49	1.30	8.99	11.79	74.25	3.17
**2015**	1.30	0.98	10.86	14.07	69.35	3.43
**2016**	0.49	1.59	11.98	6.64	76.63	2.66
**2017**	0.56	2.09	9.94	5.35	77.17	4.88
**2018**	1.17	1.85	8.87	5.80	74.98	7.33
**Average**	0.99	1.86	11.31	10.02	68.79	7.02

**Table 9:** Worked hours and lost workdays per size in OP (%).

	**Worked Hours**					
**Year**	**1–9**	**10–19**	**20–49**	**50–99**	**100–499**	**≥500**
**2009**	22.54	28.88	26.09	13.13	9.35	
**2010**	28.00	26.86	22.36	13.09	9.69	
**2011**	27.83	25.65	22.34	13.56	10.62	
**2012**	29.31	24.79	22.46	12.20	11.24	
**2013**	30.58	23.96	24.08	8.31	13.07	
**2014**	29.82	23.20	24.81	11.36	10.81	
**2015**	29.05	23.45	24.00	14.15	9.36	
**2016**	27.65	23.48	24.77	12.97	11.13	
**2017**	26.63	24.01	23.51	14.38	11.47	
**2018**	25.58	25.31	21.96	14.18	12.97	
**Average**	27.39	25.33	23.69	12.79	10.79	
**Workdays Lost**						
**Year**	**1–9**	**10–19**	**20–49**	**50–99**	**100–499**	**≥500**
**2009**	29.40	25.54	29.19	9.03	6.73	
**2010**	23.87	29.25	31.18	7.66	7.86	
**2011**	29.97	24.93	25.17	8.70	11.16	
**2012**	34.72	27.63	22.15	6.65	8.85	
**2013**	37.48	28.78	20.48	7.45	5.77	
**2014**	41.77	20.22	23.26	4.58	10.05	
**2015**	29.23	22.30	24.30	12.31	11.87	
**2016**	35.72	24.77	29.78	5.87	3.70	
**2017**	38.39	28.74	21.13	4.86	6.66	
**2018**	38.76	25.96	23.16	4.96	6.36	
**Average**	33.04	25.88	25.54	7.42	7.95	

**Table 10:** Worked hours and lost workdays per size in PP (%).

	**Worked Hours**					
**Year**	**1–9**	**10–19**	**20–49**	**50–99**	**100–499**	**≥500**
**2009**	23.57	14.75	14.18	7.97	15.72	23.80
**2010**	18.60	17.98	15.64	10.42	14.32	23.04
**2011**	17.96	16.82	15.42	10.81	14.56	24.42
**2012**	17.68	15.38	15.24	8.82	14.61	28.27
**2013**	17.40	13.83	14.07	12.12	13.27	29.32
**2014**	17.18	13.43	14.86	6.77	4.91	42.86
**2015**	18.20	14.95	16.08	9.92	11.14	29.71
**2016**	16.35	14.45	15.67	8.64	6.85	38.05
**2017**	15.70	14.68	14.91	8.96	2.09	43.66
**2018**	15.72	16.00	14.54	9.20	7.64	36.90
**Average**	17.94	15.30	15.06	9.38	10.76	31.56
**Workdays Lost**						
**Year**	**1–9**	**10–19**	**20–49**	**50–99**	**100–499**	**≥500**
**2009**	18.41	15.97	24.10	21.28	15.42	4.81
**2010**	20.62	18.65	24.92	15.76	16.86	3.19
**2011**	21.54	16.81	23.27	17.96	16.34	4.07
**2012**	20.65	18.43	16.43	19.12	20.80	4.56
**2013**	19.35	16.47	24.76	18.96	17.92	2.55
**2014**	21.26	15.99	17.45	16.94	23.12	5.25
**2015**	28.54	14.99	12.24	11.60	25.08	7.55
**2016**	21.95	15.50	27.10	12.61	16.63	6.21
**2017**	26.08	11.55	26.67	12.12	15.17	8.40
**2018**	23.06	18.51	22.31	11.83	14.89	9.40
**Average**	21.96	16.40	22.18	16.02	17.92	5.51

**Table 11:** Severity risk index per size and each scenario.

**UG**						
**Year**	**1–9**	**10–19**	**20–49**	**50–99**	**100–499**	**≥500**
**2009**	10.53	4.38	4.27	2.44	1.17	0.35
**2010**	2.58	3.48	5.42	2.94	1.59	0.23
**2011**	0.65	1.28	3.45	0.97	2.03	0.20
**2012**	2.12	1.83	3.20	2.42	2.05	0.07
**2013**	1.40	2.57	5.81	2.70	2.39	0.06
**2014**	1.02	1.14	1.36	4.48	1.26	0.10
**2015**	4.09	1.02	3.57	4.75	2.08	0.06
**2016**	3.33	1.05	4.58	2.74	3.98	0.04
**2017**	7.74	1.52	3.75	4.37	4.19	0.06
**2018**	4.01	1.31	3.01	4.07	4.33	0.10
**Average**	3.13	1.75	3.49	2.84	2.08	0.12
**OP**						
**Year**	**1–9**	**10–19**	**20–49**	**50–99**	**100–499**	**≥500**
**2009**	1.30	0.88	1.12	0.69	0.72	
**2010**	0.85	1.09	1.39	0.59	0.81	
**2011**	1.08	0.97	1.13	0.64	1.05	
**2012**	1.18	1.11	0.99	0.55	0.79	
**2013**	1.23	1.20	0.85	0.90	0.44	
**2014**	1.40	0.87	0.94	0.40	0.93	
**2015**	1.01	0.95	1.01	0.87	1.27	
**2016**	1.29	1.06	1.20	0.45	0.33	
**2017**	1.44	1.20	0.90	0.34	0.58	
**2018**	1.51	1.03	1.05	0.35	0.49	
**Average**	1.21	1.02	1.08	0.58	0.74	
**PP**						
**Year**	**1–9**	**10–19**	**20–49**	**50–99**	**100–499**	**≥500**
**2009**	0.78	1.08	1.70	2.67	0.98	0.20
**2010**	1.11	1.04	1.59	1.51	1.18	0.14
**2011**	1.20	1.00	1.51	1.66	1.12	0.17
**2012**	1.17	1.20	1.08	2.17	1.42	0.16
**2013**	1.11	1.19	1.76	1.56	1.35	0.09
**2014**	1.24	1.19	1.17	2.50	4.71	0.12
**2015**	1.57	1.00	0.76	1.17	2.25	0.25
**2016**	1.34	1.07	1.73	1.46	2.43	0.16
**2017**	1.66	0.79	1.79	1.35	7.24	0.19
**2018**	1.47	1.16	1.53	1.29	1.95	0.25
**Average**	1.22	1.07	1.47	1.71	1.67	0.17

## Data Availability

All the results of this study have been obtained from the data on occupational accidents in the Spanish mining sector, subministered by the Spanish Ministry of Labor and Social Economy.
